# Artificial Intelligence in Anaerobic Digestion: A Review of Sensors, Modeling Approaches, and Optimization Strategies

**DOI:** 10.3390/s25226961

**Published:** 2025-11-14

**Authors:** Milena Marycz, Izabela Turowska, Szymon Glazik, Piotr Jasiński

**Affiliations:** Faculty of Electronics, Telecommunications and Informatics, and Advanced Materials Centre, Gdańsk University of Technology, 80-233 Gdańsk, Poland; s199183@student.pg.edu.pl (I.T.); s199082@student.pg.edu.pl (S.G.); piotr.jasinski@pg.edu.pl (P.J.)

**Keywords:** anaerobic digestion, artificial intelligence, machine learning, soft sensors, data preprocessing, explainable AI, digital twin, process optimization, feature engineering

## Abstract

**Highlights:**

**What are the main findings?**

- Artificial intelligence (AI) and machine learning (ML) have the potential to significantly enhance the forecasting and optimization of anaerobic digestion (AD) processes. They enable efficient handling of nonlinear and multidimensional data, enhancing process control and predictive accuracy.
- Soft sensors integrating electrochemical, microbial, optical, and hybrid systems provide adaptive, real-time process control and stability monitoring in anaerobic fermentation.

**What is the implication of the main finding?**

- Further development toward autonomous and intelligent AD systems requires the creation of more reliable sensors, standardization of open-access datasets, and improvement of AI model interpretability.
- Advancing these areas will enable more predictive, transparent, and efficient biogas production, supporting the circular economy and energy transition goals.

**Abstract:**

Anaerobic digestion (AD) is increasingly recognized as a key technology for renewable energy generation and sustainable waste management within the circular economy. However, its performance is highly sensitive to feedstock variability and environmental fluctuations, making stable operation and high methane yields difficult to sustain. Conventional monitoring and control systems, based on limited sensors and mechanistic models, often fail to anticipate disturbances or optimize process performance. This review discusses recent progress in electrochemical, optical, spectroscopic, microbial, and hybrid sensors, highlighting their advantages and limitations in artificial intelligence (AI)-assisted monitoring. The role of soft sensors, data preprocessing, feature engineering, and explainable AI is emphasized to enable predictive and adaptive process control. Various machine learning (ML) techniques, including neural networks, support vector machines, ensemble methods, and hybrid gray-box models, are evaluated for yield forecasting, anomaly detection, and operational optimization. Persistent challenges include sensor fouling, calibration drift, and the lack of standardized open datasets. Emerging strategies such as digital twins, data augmentation, and automated optimization frameworks are proposed to address these issues. Future progress will rely on more robust sensors, shared datasets, and interpretable AI tools to achieve predictive, transparent, and efficient biogas production supporting the energy transition.

## 1. Introduction

Energy policy in Europe increasingly focuses on climate change mitigation and energy security. In 2022, the European Commission launched the REPowerEU plan, which explicitly aims to reduce the European Union’s reliance on imported fossil fuels, particularly natural gas from Russia. A central component of this plan is the large-scale deployment of biomethane produced from renewable and waste-based sources, such as agricultural residues, industrial by-products, waste gases, and municipal wastewater [1,2,3]. Anaerobic digestion (AD) links waste management with the production of biogas, which can be used as a renewable energy carrier. AD is a multi-stage microbial process in which organic substrates are decomposed in the absence of oxygen, yielding primarily methane and carbon dioxide (CO_2_), along with small fractions of other gases such as hydrogen sulfide and ammonia. The resulting biogas can be upgraded to biomethane and injected into natural gas grids, while the residual digestate constitutes a nutrient-rich soil amendment [4]. In addition to its environmental benefits, AD contributes to the circular economy by valorizing diverse organic residues such as food waste, wastewater sludge, and agricultural by-products [5,6].

In practice, AD operation often faces instability. The microbial consortia responsible for each metabolic stage are highly sensitive to environmental fluctuations, and process stability is easily disrupted by variations in feedstock composition, accumulation of inhibitors, or inadequate control of operational parameters such as temperature, pH, organic loading rate (OLR), and hydraulic retention time (HRT) [7,8]. Instability can result in reduced methane yields, process inhibition, or even complete reactor failure. Traditional control strategies, relying on a limited set of sensors and linear control loops, often lack the predictive power necessary to anticipate such disturbances [9]. Consequently, the optimization of AD requires new approaches that combine high-resolution monitoring with advanced analytical and decision-support tools.

Artificial intelligence (AI) and machine learning (ML) have been applied to AD in recent studies. These approaches use historical and real-time data to model nonlinear dynamics that conventional mechanistic models cannot capture. Neural networks, recurrent networks, adaptive neuro-fuzzy systems, and ensemble methods have all been applied to predict biogas yields, detect anomalies, and optimize parameters [5,10,11,12,13,14]. Hybrid gray-box frameworks combine the structure of models such as ADM1 with data-driven flexibility, offering more practical predictive capacity [15,16,17,18].

The modeling of AD processes has traditionally relied on three complementary approaches: mechanistically inspired, kinetic, and phenomenological models [19]. Mechanistic models, such as ADM1 and BioModel, provide a detailed biochemical and physicochemical description of system dynamics but require extensive parameterization and substrate characterization [20]. Kinetic models simplify these interactions using empirical rate equations, enabling faster computation but with limited generalizability [21]. Phenomenological models, in turn, focus on reproducing observed input–output relationships without explicit mechanistic detail, offering flexibility at the cost of interpretability [19]. Each class of models thus presents a trade-off between accuracy, complexity, and data requirements. Optimization of AD processes has similarly evolved from deterministic approaches, which rely on gradient-based algorithms and provide reproducible, single-point solutions, to stochastic or metaheuristic methods, such as genetic algorithms, particle swarm optimization, and simulated annealing [22,23,24]. The latter are particularly useful for nonconvex, multimodal problems typical of AD but may require high computational effort and careful tuning of algorithmic parameters. In this context, AI and ML serve as a bridge between traditional modeling paradigms. They combine the adaptability of data-driven learning with the explanatory power of mechanistic frameworks, as demonstrated by recent developments in hybrid modeling and digital twin systems [19].

Another important limitation of current mechanistic and kinetic models is their inability to adequately describe foaming phenomena in anaerobic digesters [19,25]. Foam formation and stability depend on complex interactions among surface-active compounds, gas–liquid mass transfer, hydrodynamics, and microbial community composition, which remain poorly understood at the mechanistic level [26]. As a result, existing process models often fail to anticipate foaming events that can cause operational disruptions and biomass losses [27]. AI-driven frameworks may help address this gap by extracting data-driven signatures of foaming behavior from high-frequency sensor data (e.g., pressure, gas flow, or image-based measurements) [28]. Such models could support early detection and mitigation of foaming, complementing mechanistic understanding with predictive capabilities derived from real-world process data.

The aim of this review is to examine current AI-based approaches for AD, with focus on monitoring, sensor integration, optimization algorithms, and decision support. The review considers developments in biotechnology, process engineering, and computational methods, with reference to their application in biogas and biomethane production under the REPowerEU plan.

## 2. Anaerobic Digestion and the Need for Intelligent Monitoring

### 2.1. Basics of Anaerobic Fermentation Processes

In AD, organic matter is broken down stepwise by microbial consortia, with different groups performing distinct metabolic conversions. The first stage, hydrolysis, involves extracellular enzymes that depolymerize carbohydrates, proteins, and lipids into soluble monomers. These smaller molecules are then fermented during acidogenesis, producing volatile fatty acids (VFAs), alcohols, hydrogen, and CO_2_. In the subsequent step, acetogenesis, these intermediates are further oxidized to acetate, hydrogen, and CO_2_, which finally serve as substrates for methanogens in the terminal stage of methanogenesis. The efficiency of each stage depends on the activity of microbial consortia, many of which are still not fully characterized [29,30].

The composition of biogas is governed by the carbon oxidation-reduction state of the organic matter present in the feedstock, and by the type of AD process. For example, biogas from landfills is a complex mixture of methane (35–65%), CO_2_ (15–50%) and volatile organic pollutants (VOCs; <4500 mg/m^3^) with low hydrogen sulfide (H_2_S) concentrations (<200 ppm_v_). Biogas from enclosed digesters exhibits higher methane (50–70%) and H_2_S (500–20,000 ppm_v_) contents, but lower concentrations of VOCs [31,32]. The digestate contributes to nutrient recycling, which supports circular economy applications [33,34].

AD has been studied extensively with mathematical and computational models. The Anaerobic Digestion Model No. 1 (ADM1), developed by the International Water Association, represents the most widely adopted mechanistic framework. It describes the dynamics of 32 state variables through more than 100 parameters, encompassing hydrolysis kinetics, microbial interactions, and gas–liquid transfer processes [16]. Although ADM1 is widely used in research, its application at full scale is limited. It requires extensive substrate characterization, which is often impractical for heterogeneous feedstocks such as municipal solid waste or manure. Parameter calibration is highly demanding and site-specific, and the model does not easily accommodate the stochastic fluctuations inherent in real-world conditions [9,15]. Recently, more advanced mechanistic frameworks such as the BioModel have been developed, extending beyond ADM1 by incorporating biochemical reactions together with three-phase (gas–liquid–solid) physicochemical processes, as well as the influence of biological and inorganic additives and trace metal activities. These extensions allow a more comprehensive representation of substrate degradation and gas production dynamics, supported by experimental validation and parameter optimization on full-scale biogas plant data [35,36]. Such developments provide a valuable context for integrating AI, which can complement these detailed mechanistic frameworks by improving data-driven calibration, predictive accuracy, and adaptive process control.

Data-driven approaches such as ML are increasingly applied in AD. ML techniques range from artificial neural networks to ensemble methods. These techniques offer the ability to infer complex, nonlinear relationships between input parameters and process performance without requiring explicit biochemical equations [37,38]. These models have demonstrated strong predictive performance in estimating biogas yields, forecasting system instabilities, and even serving as soft sensors (i.e., inferential models emulating sensor functions; a formal definition and classification are provided in Section 3.3) for variables such as VFA concentration or ammonia, which are otherwise costly and laborious to measure directly [10,39,40]. Hybrid or so-called gray-box approaches that integrate mechanistic insights with data-driven adaptability further expand this potential, allowing simulations to benefit simultaneously from biochemical interpretability and statistical flexibility [17,18]. For instance, Ge et al. [41] proposed a modified ADM1 (M-ADM1) that integrates a support vector machine model to dynamically predict key kinetic parameters, thereby improving simulation accuracy for various biomass feedstocks.

In summary, AD technology is promising due to the ability to recover energy from spent biomass and recycle nutrients, but has strict operational constraints related to maintaining microbial communities and variability in properties of feedstock. Addressing these issues requires monitoring and control strategies that extend beyond conventional methods.

### 2.2. Challenges in Monitoring and Control

The stable operation of AD relies on monitoring parameters that are closely interrelated. Temperature plays a critical role, as microbial activity and methane production differ significantly under mesophilic and thermophilic conditions, typically around 35–40 °C for mesophilic and 55–60 °C for thermophilic operation, although the exact ranges may vary between systems and operators [42,43,44]. pH and alkalinity reflect microbial balance: methanogens grow within a narrow pH range, while the accumulation of VFAs can quickly disrupt digester stability [45,46,47]. The relationship between OLR and HRT determines whether microorganisms have enough time to process the substrate. Excessive loading or insufficient retention can lead to biomass washout or process inhibition [33,48]. VFA and ammonia concentrations serve as sensitive early indicators of imbalance, yet continuous real-time monitoring remains challenging [7,49,50]. In laboratory and pilot-scale studies, these parameters are still often measured manually. Samples are withdrawn from the reactor and analyzed in the laboratory using standard test kits or chromatography-based methods to determine VFA, ammonia, or chemical oxygen demand (COD). Although reliable, these laboratory methods are time-consuming and susceptible to operator mistakes [9]. They also miss short-term changes in digester conditions, which can lead to problems before corrective measures are applied.

Efforts to automate monitoring have increasingly turned to online sensors that can deliver continuous data streams. Their deployment in AD is, however, technically demanding. A persistent problem is biofouling: microbial films and organic deposits accumulate on sensor surfaces, distorting measurements and gradually lowering accuracy. This is particularly evident in electrochemical probes used for pH, redox potential, or ion-selective measurements, where signal drift often requires frequent recalibration [51,52,53]. Common countermeasures include self-cleaning housings, protective membranes, and automated calibration routines. These measures slow down fouling but do not remove it entirely. More recent trials with anti-adhesive or antimicrobial coatings show potential for longer stability, though their durability in digesters still needs to be confirmed [54,55,56].

Calibration of sensors poses another practical difficulty. In many studies, models are trained on relatively small datasets derived from manual sampling. Such limited input narrows the capacity of algorithms to generalize, so even minor shifts in feedstock composition may require retraining [13,57]. Sensor drift and environmental variability add further noise, reducing reproducibility [58]. A promising approach to mitigate these limitations involves the systematic creation and evaluation of large, heterogeneous training datasets, encompassing diverse sample types. Specifically, in the context of AD, samples including agricultural residues, manure, and industrial effluents are employed to capture the full range of substrate characteristics [13,59]. By providing a comprehensive representation of sample variability, such datasets enable ML models to learn the nonlinearities inherent in sensor responses and to adapt to new conditions without necessitating a complete redesign of the system [60]. These efforts are still in early stages, and their effectiveness for full-scale operation is being evaluated [13,60].

Control strategies face comparable limitations. Conventional feedback mechanisms, particularly proportional–integral–derivative (PID) controllers, are poorly suited to AD because of its strong nonlinearity, long response times, and interdependencies among parameters [61,62]. In practice, operators often rely on trial-and-error adjustments. This approach can keep reactors running but also increases the risk of inefficiency or breakdown [63].

Research has therefore moved toward data-driven control. Soft sensors estimate variables that are expensive or difficult to monitor directly, for example, VFA concentrations, by drawing on more readily available measurements [51,64]. Anomaly detection systems can recognize deviations in system behavior before they escalate into critical issues. Hybrid approaches that combine ML with optimization methods, such as genetic algorithms or desirability analysis, can then refine loading rates and retention times to maximize methane yield [18,65]. More recently, hybrid dynamic model predictive controllers incorporating Bayesian estimation have been proposed to handle inlet variability and improve methane generation efficiency in anaerobic digestion systems [66]. Moreover, the emergence of digital twins, which are virtual replicas of digesters that combine predictive modeling with real-time process data obtained from sensors, provides a powerful tool for scenario testing, early warning, and autonomous decision-making [67,68]. These tools remain under development, but they show how process control is moving beyond empirical adjustment. The reactor’s economic efficiency can be improved by implementing the SMART (Sensor-based Monitoring and Remote-control Technology) control system, whose fuzzy logic-based decision engine ensures increased biogas production and improved volume recovery rates. Using this technology allowed for a 53% increase in methane production after four months compared to the open-loop method, while methane production increased 1.6 times slower using the conventional method than the SMART system [69].

Although many monitoring and control concepts have been proposed, their validation under industrial conditions is scarce. Future work should focus on transferring laboratory-scale monitoring strategies to full-scale biogas plants and assessing their economic feasibility and robustness to environmental variability.

## 3. Sensors in Anaerobic Reactors: State of the Art

### 3.1. Electrochemical Sensors

The operation of AD depends on continuous changes in pH, redox potential, and conductivity, which are tracked with electrochemical sensors [70]. Online monitoring provides real-time data that lead to faster detection of process disturbances and advance planning of calibration or cleaning [71]. However, sensor reliability depends on multiple factors, including equipment configuration, sampling strategies, measurement frequency, and maintenance requirements [72].

Amperometric electrochemical sensors have been developed to monitor VFAs, critical intermediates that reflect AD stability. These sensors detect current signals generated by electron transfer from electroactive bacteria such as *Geobacter anodireducens*, which colonize electrode surfaces with anodic biofilms [73]. However, sensor signals are sensitive to interference. For example, fumarate can act as an electron donor, increasing current density and distorting VFA quantification, while ions such as Cu (II) or elevated total ammonia nitrogen (TAN > 200 mg/L) reduce measurement reliability [74]. Nevertheless, electrodes composed of graphite anodes and stainless-steel cathodes have achieved accuracy comparable to gas chromatography (GC), demonstrating the promise of microbial electrochemical sensors (MESe) for online VFA monitoring [75]. For example, octane measurement using microbial biosensors yields comparable results to gas chromatography, with the market value within 24.2% in terms of gas chromatographic analysis [76]. Coupling electrochemical probes with physicochemical sensors such as temperature or conductivity electrodes can improve data quality [75]. In practice, long-term use remains constrained by microbial community shifts that affect reproducibility and by fouling of anion-exchange membranes during immersion in digestate [75,77].

Microbial potentiometric sensors (MPSs) use biofilm-mediated open-circuit potentials to track redox fluctuations in digesters [78]. MPS devices are more sensitive to changes in carbon removal than dissolved oxygen (DO) or oxidation–reduction potential (ORP) sensors. Their main advantages include the catalytic activity of biofilm-associated enzymes and the capacity for biofilm regeneration of electrode surfaces and can delay the need for electrode maintenance [79]. However, MPS systems are strongly influenced by environmental conditions such as nutrient levels, temperature, and the presence of biocides. The results of the bioelectrochemical cell solution analysis (MEC/MFC) lead to a linear correlation (R^2^ = 0.99) between current fluxes (from 0.03 ± 0.01 to 2.43 ± 0.12 A/m^2^) and VFA effects (5–100 mM), further supporting the sensitivity of MPS-based systems to biochemical variations in anaerobic environments [80]. Increased internal resistance over time also degrades signal quality and reduces reliability [81]. Biofilms may harbor pathogenic microorganisms, but they can simultaneously support beneficial processes such as surface regeneration [82].

The field of bioelectrochemical sensing has grown considerably in recent years, particularly using microbial fuel cells (MFCs) and electrolytic systems that exploit electrons released during anaerobic metabolism [52]. In most cases, the sensing element consists of microbial biofilms, which directly couple biological activity to an electrical response. A notable example is their application in monitoring gas composition, including stable isotopes such as C^13^, which can shed light on the activity of methanogenic pathways [83]. Electroactive microorganisms can spontaneously colonize electrode surfaces, forming direct contact with conductive materials needed for a stable signal [84]. However, these sensors face limitations in selectivity and repeatability. Over time, microbial consortia consume multiple carbon sources, which decreases measurement precision. To counteract this effect, genetic modulation of auxotrophic strains has been proposed to enhance signal strength, improve current density, and maintain long-term sensitivity [85]. Despite such advances, reproducibility remains a major challenge, as it is difficult to consistently replicate biofilm thickness or microbial distribution under identical conditions. Environmental stressors such as antibiotics or heavy metals further compromise sensor stability, particularly in waste streams such as dairy effluents [52,86].

Recent innovations explore hybrid sensor systems, such as those combining MFCs with gas flow meters and pH probes, which expand monitoring capabilities for intermediates like acetate, an important indicator of methanogenesis stability. These hybrid designs have achieved acetate measurement deviations within 24.2% of GC reference values, highlighting their practical potential [76,87]. Additional advances, such as pH-activated dissolvable polymeric coatings, can mitigate biofouling on electrochemical electrodes, improving long-term stability [88].

Electrochemical and potentiometric sensors remain constrained by issues of longevity, reproducibility, and biofouling. Large-scale application is further limited by trade-offs between nutrient supply, microbial stability, and the effort required for maintenance [72,89].

### 3.2. Optical and Spectroscopic Sensors

In recent years, optical and spectroscopic approaches have become increasingly important tools for monitoring AD, offering an alternative to conventional electrochemical methods. These sensor systems rely on light–matter interactions such as absorption, fluorescence, or scattering to track relevant analytes, ranging from gases and solvents to microbial metabolites. Commonly applied techniques include isotope spectrophotometry, infrared spectroscopy (IR), and spectrofluorometry [90,91,92].

Optical sensors are used either at-line, where samples are taken to an external module, or on-line, where they are placed directly in the process stream. Continuous on-line monitoring is used for hydrogen concentrations, since elevated hydrogen levels not only affect CO_2_ reduction but also accelerate VFA accumulation, destabilizing digestion. Correct sensor positioning within the reactor is therefore essential for reliable performance [93].

An illustrative example of early optical sensor innovations is the fiber-optic immersion sensor employing triphenylmethane dyes to detect organic solvents in wastewater. Its performance is strongly influenced by temperature and signal noise, as elevated temperatures accelerate solvent evaporation and alter analyte diffusion into the sensor matrix. This makes thermal control a critical factor for reliable operation. The sensor also exhibits limitations for poorly water-soluble solvents, which may produce delayed or inconsistent responses, while small donor molecules such as ammonia often show negligible absorption despite their strong reactivity. In contrast, highly volatile solvents with properties distinct from water (e.g., tetrahydrofuran, ethyl acetate) generate increased partial vapor pressures, which can artificially inflate the measured signal. Despite these constraints, the fiber-optic sensor has practical advantages: recalibration is simple and rapid, since the device only requires a water rinse between measurements [94].

Biosensors have also been coupled with optical transducers. In such systems, immobilized biocatalysts placed on single waveguides or fiber bundles catalyze analyte-specific reactions, while the resulting products are recorded via fluorescence or chemiluminescence detection [92,95]. The biocatalyst thus acts as a molecular bridge between the analyte and the transducer, enabling conversion of biochemical interactions into measurable optical signals. In this approach, chlorophyll fluorescence can be used to detect algal growth. Such measurements, often performed with laboratory spectrofluorimetry under laser excitation, provide valuable information on microbial community dynamics and help prevent excessive algal proliferation that could destabilize fermentation [92,96].

Total internal reflection fluorescence (TIRF) uses lipid membranes on planar optical waveguides to control the penetration depth of the evanescent field. The angle of light coupling to the waveguide depends on its refractive index, allowing miniaturized sensors suitable for quantifying gas concentrations, refractive indices of liquids, or humidity [92,97]. A complementary technology is surface plasmon resonance (SPR) sensing, in which incident light excites surface plasmons at the metal–dielectric interface. This enables trace-level detection of heavy metals, with reported sensitivities of 10 ppm Cu and 13.76 ppm Pb [98].

Optical methods are widely used for biogas quality assessment. For example, non-dispersive infrared (NDIR) modules equipped with photoacoustic detectors provide high-resolution, real-time quantification of methane and CO_2_. In this configuration, mid-infrared light is converted into acoustic waves, with amplitudes directly proportional to analyte concentrations [90]. Additionally, NIR spectroscopy used in the AD reactor for TAN analysis showed strong model correlation (R^2^ = 0.91) and low prediction error (RMSEP = 0.32 g N/L) for the range of 1.5–5.5 g N/L. These results confirm the high efficiency of the probe during measurements in filtration mode and in assessing the quality of substrates [99]. In parallel, isotope fractionation techniques have been applied to monitor microbial responses to environmental perturbations such as salt stress [91].

In wastewater treatment applications, where membrane fouling remains a persistent challenge, UV and IR spectroscopic scattering indicators are widely applied for real-time monitoring of fouling formation [100]. Despite their advantages, including versatility, rapid response, and relatively low operational costs, optical sensors continue to face practical challenges. These include probe biofouling, calibration drift, and optical artifacts caused by air bubbles during mixing or aeration, which distort absorption signals [72,101]. Moreover, their applicability is limited for certain compounds such as carbohydrates and saturated hydrocarbons, which lack significant UV absorption and therefore require complementary analytical techniques [92,102].

Optical and spectroscopic sensors offer high-resolution, non-invasive monitoring of AD. Their wider use depends on solving problems of fouling, calibration drift, and analyte-specific limitations.

### 3.3. Sensor Integration and Data Acquisition

Monitoring in AD involves both physical sensors and data acquisition frameworks that support modeling. Online measurements of flow rates, gas composition, and auxiliary variables provide rapid, continuous insights into process dynamics, in contrast to offline sampling, which introduces delays and limits temporal resolution [13]. Online systems capture short-term fluctuations in reactors that are missed by offline sampling.

A key element in this integration is the use of soft sensors, which link direct physical measurements with mathematical modeling to estimate process variables that are otherwise difficult or expensive to quantify. Indirect indicators, such as nutrient consumption or the release of metabolic intermediates, can provide useful proxies for biomass growth and substrate turnover. These indicators supply critical data for model-based predictions. Despite the rise of advanced sensing technologies, simple titration-based assays remain common in both research and industry, largely due to their low cost and adaptability [103,104,105].

**Figure 1:**
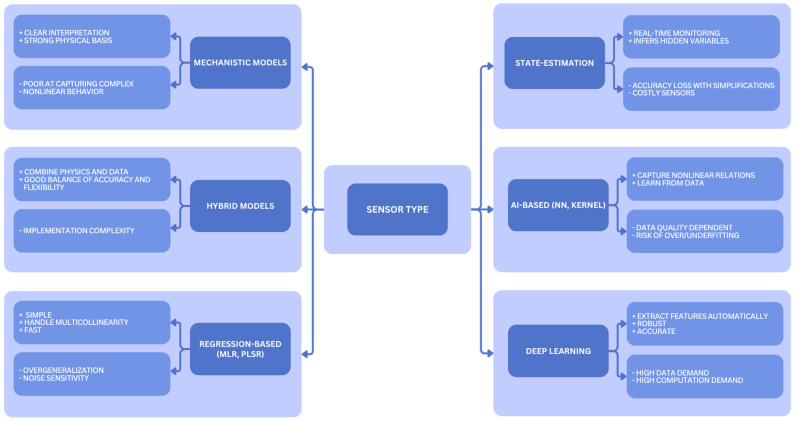
Schematic representation of soft sensor modeling strategies applied to AD processes [103,106,107,108,109,110,111,112].

Soft sensors can be classified according to the modeling strategies used to transform raw measurements and auxiliary variables into reliable process information. These strategies encompass mechanistic, regression-based, state-estimation, AI–based, and deep learning-based frameworks, as illustrated schematically in Figure 1.

**Mechanistic models** construct mathematical relationships between target and auxiliary variables based on balance equations and detailed process analysis [106]. While this approach provides interpretability and clear links to underlying bioprocesses, it struggles to capture the inherent complexity, variability, and nonlinear behavior of AD [107]. To address these limitations, hybrid models have emerged that combine mechanistic foundations with data-driven flexibility. Such models integrate physical and mathematical correlations to provide both qualitative and quantitative insights into degradation-phase dynamics and associated gas production, while also accounting for microbial growth patterns [103,108].

**Regression-based approaches** use statistical correlations between measured inputs and predicted outputs. Multiple linear regression (MLR) and partial least squares regression (PLSR) are widely applied, as they effectively handle collinearity among auxiliary variables and establish linear mappings between measured inputs and predicted outputs [103,109]. However, such models often generalize results excessively, which can reduce accuracy, obscure meaningful signal components, or introduce residual noise.

**State-estimation models** infer target variables from auxiliary measurements by approximating system behavior [107]. Such models are particularly useful for real-time monitoring, yet their accuracy can decline when system complexity is reduced to ease computational load. Moreover, both the limited precision and the expense of sophisticated instrumentation continue to hinder their broader adoption in large-scale industrial AD processes [103,109].

A conceptual distinction exists between regression-based and AI-based frameworks. Regression models, such as MLR and PLSR, rely on predefined linear or polynomial relationships between inputs and outputs. In contrast, AI-based soft sensors include ML and deep learning algorithms, such as neural networks and support vector machines, which can also perform regression but learn nonlinear and adaptive relationships directly from data. This distinction clarifies the methodological scope of each group and ensures consistency with the discussion of regression and classification tasks in Section 4.1.

Recent research increasingly explores **AI-based** soft sensors. Neural networks, for example, can establish nonlinear mappings between auxiliary and target variables by learning directly from training datasets [110]. Similarly, kernel-based approaches transform variables into higher-dimensional feature spaces, where linear algorithms can then be applied to uncover nonlinear relationships [111]. Nevertheless, both approaches face challenges, as network topology, kernel selection, and the quality of training samples strongly influence performance. Neural networks are prone to overfitting or underfitting, which may compromise generalizability [103,112]. These issues are discussed in more detail in Section 4.2.

The most advanced developments involve **deep learning–based frameworks**, which extend neural network architectures with multiple hidden layers to automatically extract informative features from raw data and capture temporal dynamics inherent to AD processes. These methods have demonstrated improved robustness and predictive accuracy under fluctuating environmental and operational conditions [103].

Despite ongoing challenges such as delays in analysis, a tendency toward over-simplification, and high implementation costs, the integration of physical sensors with AI-based data acquisition remains limited. For clarity and comparison, Table 1 outlines the principal sensor types used in AD monitoring, along with their measurable parameters, advantages, limitations, potential for AI integration, and key literature references. While numerous sensor types have been tested for AD, few studies directly compare their performance across feedstocks or operational regimes. Establishing standardized testing protocols and open databases for sensor benchmarking would facilitate more objective assessment and model integration.

## 4. AI for Anaerobic Fermentation

### 4.1. From Data to Insight: ML Paradigm

Modern sensor systems generate large datasets during AD operation. Analyzing these data requires methods able to identify patterns and predict system behavior. ML is increasingly applied in this context, as it can model nonlinear dependencies and dynamic fluctuations [65,113]. Unlike conventional control strategies, which often respond only after disturbances occur, ML-based tools support early interventions and contribute to more stable reactor performance. Nevertheless, their effectiveness depends critically on the amount, quality, and diversity of available data. This is a persistent challenge in AD, where measurements may be incomplete, affected by sensor drift, or constrained by limited sampling frequency [13,114].

Most ML work in AD uses either classification or regression tasks. Classification models are typically used to distinguish between stable and unstable operating states, detect inhibition events, or group substrates based on microbial community composition [115]. These tasks may take the form of binary (e.g., failure vs. stable operation), multiclass (different feedstock types), or multilabel classification. Regression models, by contrast, predict continuous outcomes such as methane yield, VFA concentration, or TAN. Their performance is generally evaluated using metrics such as root mean square error (RMSE), which provide a quantitative benchmark for predictive accuracy [114].

**Figure 2:**
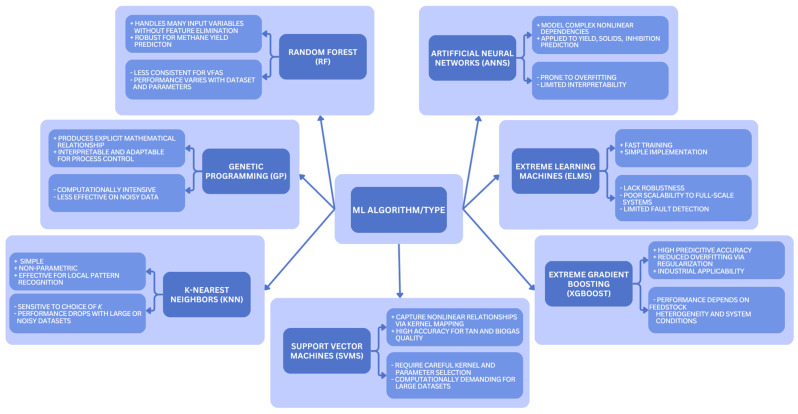
Schematic representation of ML algorithms applied to AD, illustrating major methodological categories and their interrelations [110,114,116,117,118,119,120,121,122,123].

A broad range of machine learning algorithms has been applied to AD, including tree-based ensembles such as Random Forest (RF), evolutionary approaches such as Genetic Programming (GP), instance-based methods such as k-Nearest Neighbors (KNN), kernel-based methods such as Support Vector Machines (SVMs), neural architectures such as Artificial Neural Networks (ANNs) and Extreme Learning Machines (ELMs), as well as ensemble boosting frameworks such as Extreme Gradient Boosting (XGBoost). These approaches, summarized schematically in Figure 2, differ in data requirements, interpretability, and computational complexity, providing diverse capabilities for process prediction, optimization, and fault detection. **RF** is often applied because it can process many input variables without prior feature elimination. It performs well for methane yield prediction but less consistently for VFAs. In such cases, **GP** offers greater transparency and adaptability, as it generates explicit mathematical relationships that can be integrated into process control systems [116]. Comparative studies show that RF often outperforms **KNN** in regression tasks for methane prediction, although results may vary depending on the dataset and process parameters. KNN performance is highly dependent on the choice of the k parameter and tends to suffer from reduced precision [114,117].

**SVMs** map nonlinear input data into higher-dimensional feature spaces, which enables linear separation. Beyond conventional algorithms, probabilistic approaches such as multi-task Gaussian processes (MTGPs) have recently demonstrated strong predictive performance for simultaneous estimation of biogas, soluble COD, and VFA dynamics, outperforming mechanistic AM2 models while retaining uncertainty quantification capabilities [124]. This makes them suitable for both classification and regression tasks. In AD studies, SVMs have shown high accuracy in predicting TAN values and biogas quality, often outperforming analytical methods and neural networks [118,119].

**ANNs** have also been explored, benefiting from their ability to model complex nonlinear relationships between process variables. They have been applied to predict methane yields, volatile solids concentrations, and inhibition events [110,120]. Their performance, however, depends strongly on network architecture and training strategy, and they are prone to overfitting and sensitivity to hyperparameter choices [118]. Variants such as **ELMs** set hidden layer weights randomly and calculate output weights analytically, which shortens training time. These models often lack robustness and have limited fault detection capacity, so their use in full-scale systems remains restricted despite positive laboratory results [121].

More recently, ensemble and boosting approaches have gained prominence. **XGBoost** has demonstrated industrial-scale applicability, offering improved prediction of methane production across heterogeneous feedstocks while reducing overfitting through regularization [122,123]. Reported normalized RMSE values of around 21% have been achieved, though performance still depends on feedstock heterogeneity and system conditions [123].

Algorithm choice depends on the task and data quality. Hybrid approaches that combine mechanistic models such as ADM1 with ML are being tested to balance interpretability with predictive flexibility [17,18].

Integration of AI models with sensor data is crucial for translating raw measurements into actionable insights. Different AI and ML architectures exhibit specific advantages depending on the nature of the data produced by various sensors [13,103]. For example, time-series models such as Long Short-Term Memory (LSTM) networks and Recurrent Neural Networks (RNNs) are particularly effective for continuous electrochemical or potentiometric sensor data, as they capture temporal dependencies and detect gradual signal drifts caused by fouling or process fluctuations [10,11]. In contrast, Convolutional Neural Networks (CNNs) excel in analyzing spectroscopic and optical sensor outputs, where spatial or spectral feature extraction is required to identify subtle patterns in absorbance, fluorescence or scattering data [14]. Ensemble and tree-based algorithms (e.g., Random Forest, XGBoost) are often preferred for hybrid sensor arrays combining multiple data modalities, offering robustness to noise and strong feature interpretability [125]. A concise summary of the relationship between sensor types and suitable AI/ML approaches is provided in Table 2. A more detailed comparative summary of ML algorithms applied to AD, including their specific inputs, outputs, and performance metrics, can be found in the recent comprehensive review by Murali et al. [126].

Despite the good results of hybrid and ensemble models, there are very few databases for training models in AD. Developing such databases for regions or countries, for example, would greatly facilitate model testing, training, and validation.

### 4.2. Training–Validation–Deployment: Practical Workflow

**Figure 3:**
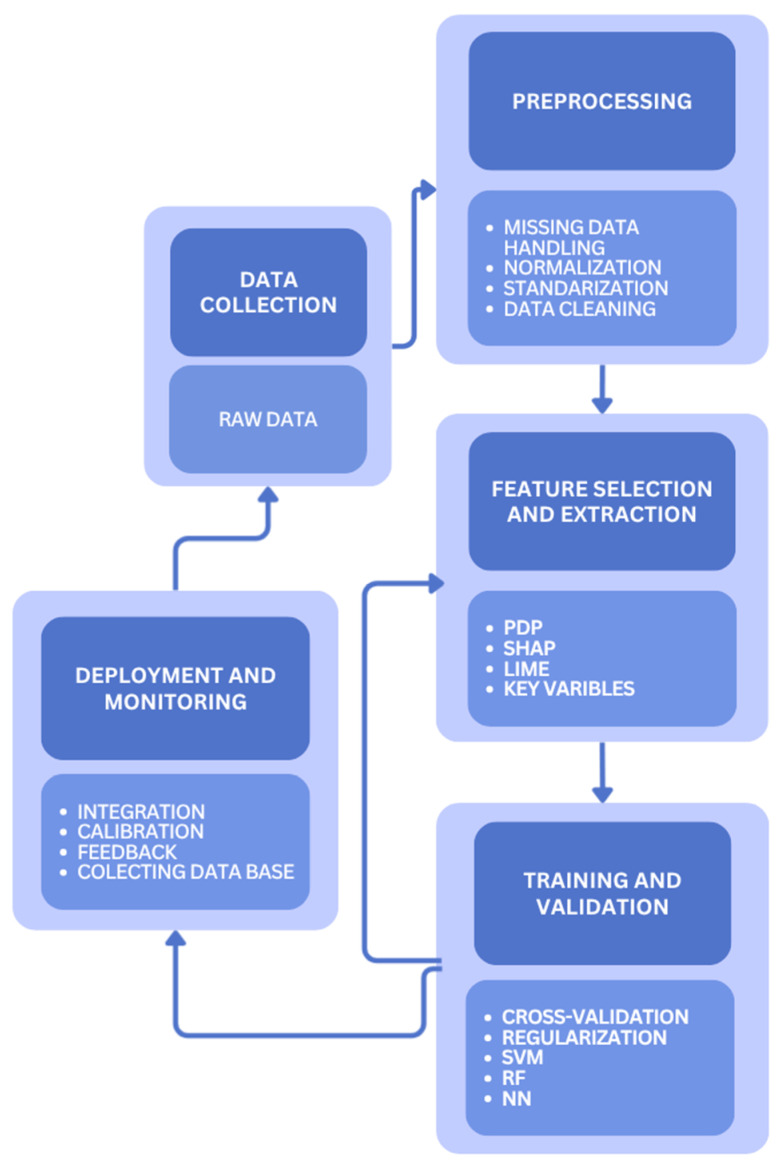
Schematic workflow of ML model development and deployment for AD processes.

Applying ML in AD involves several stages: data collection, preprocessing, model training, validation, and deployment. Each stage affects the reliability of predictions. Moreover, they present specific challenges in the AD context and determines the reliability of final model predictions [13,58]. These sequential steps, illustrated schematically in Figure 3, form the practical workflow for developing, validating, and implementing ML models in AD.

The workflow begins with data preparation. Raw measurements must be cleaned, normalized, and formatted to match ML model requirements. Missing values, common in both laboratory and industrial datasets due to sensor drift, transmission errors, or biofouling, must be carefully addressed. In practice, sensor biofouling and signal drift frequently result in data that are not missing at random (NMAR), complicating imputation and increasing the risk of biased model training if not properly handled. Their treatment depends on the mechanism of missingness: missing completely at random (MCAR), missing at random (MAR), or NMAR [130,131]. Two main strategies are applied: (i) imputation, which estimates missing values using statistical or ML methods (e.g., regression, hot-deck, or multiple imputation), and (ii) deletion, which removes affected entries either entirely (listwise) or selectively (pairwise) [130,131,132]. More specifically, imputation refers to estimating missing values based on observed data. Simple approaches include mean or median substitution [133]. More advanced strategies, such as regression imputation, involve first building a regression model on available data and then predicting missing values [58]. Regression-based approaches preserve dataset size but may require large samples for reliability. Another commonly used method is hot-deck imputation, in which donor values from similar cases are substituted for missing entries. Variants include random, nearest-neighbor, or sequential hot-deck procedures [134]. While this maintains correlations within the dataset, random donor selection may lead to inconsistent substitutions [135]. The alternative is deletion, i.e., explicit removal of incomplete records. Listwise deletion eliminates entire observations containing any missing values and is considered reliable only under MCAR conditions, as it risks discarding valuable information when missingness is systematic or datasets are small [136]. Pairwise deletion, in which only the missing values are excluded from specific analyses, is more accurate for MCAR or small amounts of MAR data, but it is time-consuming and may still reduce regression stability [58,137].

Additionally, some ML algorithms particularly decision tree models such as Random Forest can natively handle missing data without requiring explicit imputation or deletion. These models utilize internal mechanisms such as “surrogate splits”, which are alternative decision paths based on correlated features that are used in the case of a missing value. This preserves model consistency and reduces errors resulting from data deletion. Although this functionality is rarely used in AD modeling, it provides an interesting alternative to pairwise deletion, which can lead to inconsistent or biased statistical results [138,139].

Beyond handling missing data, preprocessing also involves data normalization and standardization, particularly important when combining measurements from different sources [114,140]. Common approaches include Z-score normalization, which rescales values based on deviation from the mean:

(1) $$z=\frac{\left(X\;-\;\mu \right)}{\sigma },$$

where X is the observed measurement, μ the dataset means, and σ the standard deviation. Z-score normalization is fast, reduces the influence of outliers, and facilitates comparison across variables, though it requires datasets to be first cleaned of missing values [134,141,142]. Another widely used method is min–max normalization, which linearly transforms data into the range 0–1:

(2) $$v=\frac{v^{\prime }\;-\;min}{max\;-\;min}\left(1\;-\;0\right)+0$$

where v′ is the raw value, and min and max are the minimum and maximum values of the dataset. This method is intuitive and transparent, and while it reduces the effect of outliers, it is sensitive to extreme values [130,142].

Once preprocessed, datasets are typically divided into training, validation, and test subsets. The training set is used to optimize model parameters, the validation set provides feedback during optimization to avoid overfitting, and the test set offers an unbiased assessment of generalization performance. Metrics such as RMSE, coefficient of determination (R^2^), or classification accuracy are commonly used to benchmark predictive performance [143].

Feature selection is equally important, as it identifies the most relevant subset of variables, thereby improving prediction accuracy, reducing noise, and simplifying computations. In AD, commonly selected features include pH, temperature, COD, oxidation–reduction potential (ORP), dissolved oxygen (DO), total solids (TS), ash content, volatile solids (VS), and TAN [131,132]. Moreover, process indicators in AD such as pH, COD, TAN, VFAs, or temperature are often highlighted as strong predictors of methane yield and system stability [114,136,140]. Feature selection reduces noise and the so-called “black box” problem, limits overfitting, and highlights variables that most affect model predictions [143,144]

The “black box” issue arises when models such as neural networks or random forests produce accurate predictions but lack transparent internal mechanisms, limiting reproducibility and user trust. To address this, feature importance evaluation methods (e.g., SHAP, LIME, and partial dependence plots (PDPs)) can be applied, enabling insights into how individual variables contribute to model output [122,144].

During model development, two algorithmic pitfalls must be addressed: overfitting, where models adapt too closely to the training data, and underfitting, where they fail to capture relevant patterns even in training. Strategies such as cross-validation, regularization, and ensemble learning are employed to mitigate these risks [142,145]. Neural networks and deep learning architectures, for example, can model highly nonlinear interactions, but they require careful tuning of network depth, neuron numbers, and stopping criteria to avoid overfitting [134,146].

In the context of AD, overfitting is a particularly significant issue when models are trained on data collected under narrowly defined conditions—for instance, using a limited number of substrates, reactor types, or data from a single facility operating under stable conditions. In such cases, the model learns only a restricted range of patterns and loses accuracy when applied to data outside the training domain. This is especially important because feedstock composition, temperature, and microbial community structure can vary significantly between biogas plants, leading to extrapolation errors. To mitigate these issues, robust validation strategies and advanced approaches such as reinforcement learning can be employed to improve model generalization and adaptability [13].

In deployment, trained models are integrated with operational platforms such as soft sensors, digital twins, or supervisory control systems. In this phase, continuous monitoring and recalibration are critical, since the statistical properties of incoming data often differ from those of training datasets due to feedstock variability, seasonal changes, or sensor degradation [135]. Deployment strategies must also balance accuracy with practicality: while deep learning models can achieve high precision, simpler methods such as RF or SVMs may be more suitable for real-time or on-site use because of lower computational requirements and higher interpretability [13,58,122]. Dynamic feed scheduling frameworks have also been developed, integrating predictive control and optimization to adjust feeding patterns in response to changing market and process conditions, leading to improved operational flexibility and economic returns [147].

Current ML workflows in AD still lack standardized pipelines for data preprocessing, feature selection, and validation across different systems. Developing harmonized procedures would improve reproducibility and enable fair comparison between algorithms used for AD monitoring and optimization.

## 5. Practical Considerations and Research Gaps

### 5.1. Sensor Limitations and Maintenance Issues

Monitoring AD processes depends on sensor accuracy, but the devices currently in use have multiple operational weaknesses. Electrochemical sensors, such as pH electrodes, are widely used but require frequent calibration and surface cleaning to maintain accuracy. Their long-term stability depends on implementing dedicated cleaning strategies, including hydraulic, mechanical, chemical, or ultrasonic methods, that extend the maintenance-free lifespan of electrodes. Integrating fully automated cleaning and recalibration interfaces can further reduce operational effort while improving data reliability [50,148,149]. Optical sensors degrade more slowly under fouling or surface contamination and usually require less maintenance. However, electrochemical configurations can be enhanced with advanced functionalities, such as automated impedance checks of membranes and glass electrodes, enabling earlier fault detection and minimizing downtime [50,150].

Normalization procedures are also critical in sensor calibration. For example, IR and colorimetric sensor arrays often employ normalization to predict component concentrations in previously unmeasured samples. A multidimensional VFA detection platform using a 23-dye matrix has been tested; it shortens sample interaction time and lowers operational costs [151].

Optical sensors are increasingly used in AI-based monitoring, as their signals can be integrated with automated data processing. Their uptake reflects limitations of electrochemical devices and the need for more stable input to soft-sensor models.

### 5.2. Data Scarcity and the Need for Public Datasets

Data availability is a major limitation for applying AI in AD, since obtaining sufficient high-quality samples is difficult. Collecting sufficient high-quality samples is particularly difficult, as invasive sampling can disturb fermentation processes. Online sensors partially mitigate this issue, but training robust soft-sensor models still requires large, diverse datasets. Generative adversarial networks (GANs) have been tested to artificially expand training datasets and compensate for limited sampling [103].

ML methods depend on large, reliable datasets to describe nonlinear relations between process variables and system behavior. However, the high costs and complexity of experimental work often result in sparse and noisy datasets. This limitation reduces model generalizability and increases retraining demands [152]. Open experimental and computational datasets can improve reproducibility and make it possible to compare results across studies [153].

Open datasets also introduce certain challenges. Studies show that neural network performance can be negatively affected by data preprocessing steps such as inconsistent formatting, scaling, or normalization procedures applied across different sources. Methods such as dropout regularization, sample normalization, transfer learning, and pretraining are used to improve model performance when data are limited [152]. Selecting the most effective strategy remains difficult due to the lack of systematic comparisons between available methods [154]. A recent study combined explainable ML with detailed microbial and chemical fingerprinting, identifying taxa such as *Oscillibacter* and *Clostridium sensu stricto* as potential biomarkers of reactor stability [155].

AI also holds potential beyond monitoring by supporting experiment planning and design. In highly dynamic and nonlinear AD processes, predictive models can inform both maintenance scheduling and process optimization. Achieving this, however, requires significantly larger datasets coupled with improved sensor technology, capable of capturing correlations between key variables. Larger datasets combined with improved sensors are needed to capture correlations between key variables and to develop more reliable predictive models [7].

Process stability depends on better data acquisition and improved sensors. Combined with statistical and AI-based methods, AD operations can shift toward predictive, resilient, and resource-efficient process management.

## 6. Conclusions and Future Perspectives

AI is gaining importance as a tool for advancing AD. Recent developments in ML show that data-driven models can forecast methane production, detect early signs of process disturbances, and support operational decisions in ways that exceed the capabilities of conventional approaches [7,65,122]. When combined with emerging sensor technologies and soft-sensor concepts, AI has the potential to make AD more predictive, resilient, and resource-efficient, in line with the principles of the circular economy and the ongoing energy transition.

Despite this promise, industrial application of AI in AD faces several hurdles. The main obstacles to large-scale implementation are the scarcity of high-quality, standardized datasets, the sensitivity of ML algorithms to hyperparameter selection, and the limited interpretability of most models. Harmonized protocols for data acquisition, formatting, and sharing would improve reproducibility and allow benchmarking across laboratories and scales [13,58]. Addressing hyperparameter sensitivity will require automated and adaptive optimization strategies that reduce reliance on expert knowledge [58,112]. Equally important, explainable AI approaches such as feature importance analyses and interpretable ensemble models could help overcome the “black-box” barrier to industrial and regulatory acceptance [58,118].

To move in this direction, several areas of research require attention. One concerns the hardware layer: more robust sensors and the integration of heterogeneous data streams are essential to establish a reliable foundation for AI-based monitoring. Another priority is algorithmic: hybrid gray-box models that merge mechanistic understanding with the flexibility of ML could help reconcile accuracy with interpretability [17,18]. At the system level, digital twins and autonomous control architectures offer a route toward self-optimizing, full-scale biogas plants. However, the practical implementation of Industrial-scale Digital Twins still faces challenges related to real-time data synchronization, computational scalability, and model validation under dynamic operating conditions. Establishing standardized interfaces between sensor networks, control systems, and simulation platforms will be essential for their large-scale deployment [156,157,158]. Another emerging trend is the use of Reinforcement Learning (RL) for developing adaptive and autonomous control strategies in AD. RL algorithms can continuously learn from plant feedback to optimize operational parameters such as feedstock loading or temperature, enabling truly self-optimizing systems [159,160]. Preliminary studies have shown that RL can enhance process stability while reducing the need for manual supervision [161]. In parallel, Federated Learning (FL) has emerged as a promising paradigm to address data scarcity and privacy concerns. By enabling collaborative model training across multiple facilities without sharing raw data, FL can help build more generalized and transferable AI models while preserving data confidentiality [162,163]. Advancing these developments will depend on close collaboration between biotechnology, computational science, and process engineering, as well as stronger partnerships between academia and industry.

AI should not be seen as a universal solution, yet it can play a decisive role in optimizing AD. Advancement in three areas, data standardization, model transparency, and sensor integration, will determine how quickly AI applications move from proof-of-concept studies to routine industrial practice. Meeting these requirements would make it possible to achieve more stable, efficient, and scalable biomethane production, directly supporting the goals of REPowerEU and the broader energy transition.

## Figures and Tables

**Table 1:** Comparison of sensor types for AD monitoring.

Sensor Type	Main Parameters Measured	Advantages	Limitations	Potential for AI Integration	References
**Electrochemical (pH, ORP, EC)**	pH, redox potential, conductivity, VFAs	Low cost, continuous monitoring, established technology	Biofouling, calibration drift, interference from inhibitors	Moderate—requires preprocessing	[52,70]
**Microbial Electrochemical (MESe)**	VFAs, acetate, redox changes	High sensitivity, direct microbial interaction	Sensitive to ammonium, heavy metals, biofilm variability	High—nonlinear signals suitable for ML	[74,75]
**Microbial Potentiometric (MPS)**	Redox potential, organic load indicators	Real-time monitoring, low maintenance due to biofilm regeneration	Signal instability, internal resistance, variability in biofilms	High—dynamic signals useful for AI	[78,81]
**Optical (IR, NDIR, fluorescence)**	Gas composition (CH_4_, CO_2_, H_2_), isotope fractionation, microbial activity	Non-invasive, rapid response, minimal sample preparation	Calibration instability, interference from bubbles/fouling	Very high—direct integration with ML models	[90,91,100]
**Spectroscopic (SPR, TIRF)**	Heavy metals, refractive index changes	High precision, compact sensors, wide application potential	Complex setup, sensitive to fouling and environmental noise	High—suitable for feature extraction	[97,98]
**Hybrid and Integrated Systems**	Multiparameter (pH, gas flow, acetate, VFAs)	Complementary measurements, improved accuracy	Longevity, complex maintenance, storage/nutrient conditions	Very high—multiparameter datasets support AI	[76,87,88]

**Table 2:** Representative AI and ML models suited for different sensor data types in AD monitoring.

Sensor Type	Typical Data Characteristics	Most Suitable AI/ML Models	Advantages of Integration	References
**Electrochemical/Potentiometric**	Continuous time-series signals (pH, ORP, conductivity, VFAs)	LSTM, RNN, GRU ^1^	Capture temporal dependencies; effective for drift detection and anomaly prediction	[13]
**Optical/Spectroscopic**	High-dimensional spectral or image-like data (IR, NDIR, fluorescence)	CNN, Autoencoders	Extract spatial/spectral features; robust to signal noise and wavelength variability	[103]
**Hybrid/Multi-sensor systems**	Heterogeneous, multimodal datasets (electrochemical + optical + microbial)	Random Forest, XGBoost, Hybrid ANN–GA ^2^	Handle mixed data types; enable feature selection and model interpretability	[125,127]
**Microbial/Bioelectrochemical**	Nonlinear, low-frequency signals with stochastic variability	ANFIS ^3^, SVM, Hybrid ANN	Manage nonlinearities; suitable for pattern recognition in noisy environments	[128,129]

^1^ GRU—Gated Recurrent Unit. ^2^ Hybrid ANN–GA—Hybrid Artificial Neural Network combined with Genetic Algorithm. ^3^ ANFIS—Adaptive Neuro-Fuzzy Inference System.

## Abbreviations

AD	Anaerobic Digestion
AI	Artificial Intelligence
ML	Machine Learning
ANN	Artificial Neural Network
SVM	Support Vector Machine
RF	Random Forest
KNN	k-Nearest Neighbors
GP	Genetic Programming
OLR	Organic Loading Rate
HRT	Hydraulic Retention Time
VFAs	Volatile Fatty Acids
TAN	Total Ammonia Nitrogen
COD	Chemical Oxygen Demand
TS	Total Solids
VS	Volatile Solids
ORP	Oxidation–Reduction Potential
DO	Dissolved Oxygen
ADM	Anaerobic Digestion Model
PID	Proportional–Integral–Derivative Controller
MESe	Microbial Electrochemical Sensor
MPS	Microbial Potentiometric Sensor
MFC	Microbial Fuel Cell
NDIR	Non-Dispersive Infrared
IR	Infrared Spectroscopy
UV	Ultraviolet Spectroscopy
SPR	Surface Plasmon Resonance
TIRF	Total Internal Reflection Fluorescence
PLSR	Partial Least Squares Regression
MLR	Multiple Linear Regression
GAN	Generative Adversarial Network
XGBoost	Extreme Gradient Boosting
RMSE	Root Mean Square Error
R^2^	Coefficient of Determination
VOCs	Volatile Organic Compounds
